# THE RELATIONSHIP OF THE TRAJECTORY OF COMORBIDITY ON FUNCTIONAL STATUS IN A COMMUNITY SAMPLE OF ELDERS

**DOI:** 10.1093/geroni/igz038.3046

**Published:** 2019-11-08

**Authors:** Carl Pieper, Barrett Bowling, Gerda Fillenbaum

**Affiliations:** 1 Duke University Medical Center, Durham, North Carolina, United States; 2 Duke University Medical Center, Department of Medicine, Durham, North Carolina, United States; 3 Duke University Medical Center, Center on Aging, Durham, NC, United Arab Emirates

## Abstract

The Duke Established Populations for the Epidemiologic Study of the Elderly (EPESE) study is a 1:7 random sample elderly over 65YO at baseline living in the Piedmont NC, followed yearly for 10 years beginning in1986. There were 4162 participants at baseline of which slightly over 1100 were still alive and responding at year 10. The participants self-reported on 5 comorbidities (cancer, high BP, diabetes, stroke and heart attack) at each year, as well as 4 functional scales - Katz, Rosow-Breslau, Nagi, and IADL scales. Using Mixed Models, the slope (trajectory) and intercept (level) of comorbidity over 10 years was estimated for each person, and correlated with the function scales in the final year. Correlations of the final comorbidity trajectory with the 4 functions levels ranged between 0.12 and 0.18, the trajectory with function ranged between 0.15 and 0.27. All p-values<0.0001. The first Canonical Correlate of function with comorbidity was 0.28 (p<0.0001). Slope and intercept were related jointly to each outcome and multivariately (p<0.001 for the joint effects, controlling for race, gender, age, and years of education. The multimorbidity relationship with function differed by race (omnibus p-value for the raceXmultimorbidity interaction p=0.03 (by Wilk’s Lambda), controlling for the demographics). This method is an example of combining longitudinal and cross-sectional outcomes and shows that the change in number of comorbidities is associated with functional status level.

